# Diagnosis of isolated hepatic tuberculosis in an immunocompetent individual: a case report and review of misdiagnosed cases

**DOI:** 10.3389/fmed.2025.1687127

**Published:** 2025-11-14

**Authors:** Lei Li, Jianjun Zhang, Qitai Song, Lingxia Cheng

**Affiliations:** 1 Emergency Department, Zigong Fourth People’s Hospital, Zigong, China; 2 Emergency Department, Second Affiliated Hospital of Chongqing Medical University, Chongqing, China; 3 Emergency Department, Affiliated Hospital of Southwest Medical University, Luzhou, China

**Keywords:** *Mycobacterium tuberculosis*, hepatic tuberculosis, misdiagnosis, carcinoma, early biopsy

## Abstract

**Introduction:**

Hepatic tuberculosis is a rare disease entity even in endemic areas of *Mycobacterium tuberculosis*. It is usually accompanied by pulmonary tuberculosis or enteric tuberculosis. Due to the non-specific clinical symptoms and imaging features may lead to misdiagnosis and even unnecessary surgery.

**Case presentation:**

A 44-year-old woman presented with complaints of abdominal pain for the past 2 months. The patient was diagnosed with a solitary hepatic tuberculosis abscess that was identified by abdominal CT and liver biopsy. The patient was started on anti-tuberculous therapy (isoniazid, rifampin, ethambutol, pyrazinamide). One month after diagnosis, an abdominal ultrasound showed the abscess had enlarged to approximately 4.4 cm × 3.4 cm, with heterogeneous echogenicity and internal septations. The patient subsequently went to a specialist hospital for further treatment. Telephone follow-up revealed that the patient continued anti-tuberculosis treatment, with symptoms gradually improving and essentially resolving after 6 months.

**Conclusion:**

Hepatic tuberculosis is relatively rare in clinical practice and can easily be misdiagnosed as hepatocellular and cholangiocellular carcinoma, leading to unnecessary surgery. Postoperative pathological examination confirms the diagnosis of hepatic tuberculosis. A solitary tuberculous liver abscess creates a clinical barrier for treating physicians. We summarized cases that have been misdiagnosed as tumors over the past 20 years. When encountering suspicious patients clinically, it is important to inquire in-depth about the history of tuberculosis and to perform an ultrasound-guided liver lesion biopsy for pathological examination as early as possible to achieve early diagnosis and treatment.

## 1 Introduction

Tuberculosis (TB) is a global health issue with particularly high prevalence in developing countries. The global burden of tuberculosis was estimated to be 10.6 million cases in 2022 (1). Abdominal tuberculosis is one of the most common extrapulmonary diseases (2). The incidence of liver involvement is less than 1% and it is usually associated with pulmonary or gastrointestinal infection foci (3). Primary hepatobiliary tuberculosis is considered very rare in abdominal tuberculosis. It is sporadically reported in the literature. Hepatic tuberculosis lacks typical clinical manifestations and may be difficult to distinguish from other malignant tumors, such as hepatocellular carcinoma, intrahepatic cholangiocarcinoma, cholangiocarcinoma, and liver abscesses. Hepatic tuberculosis may lead to misdiagnosis and even unnecessary surgery. Here, we report a case of a solitary tuberculous liver abscess and review the relevant literature.

## 2 Case summary

**Figure 1:**
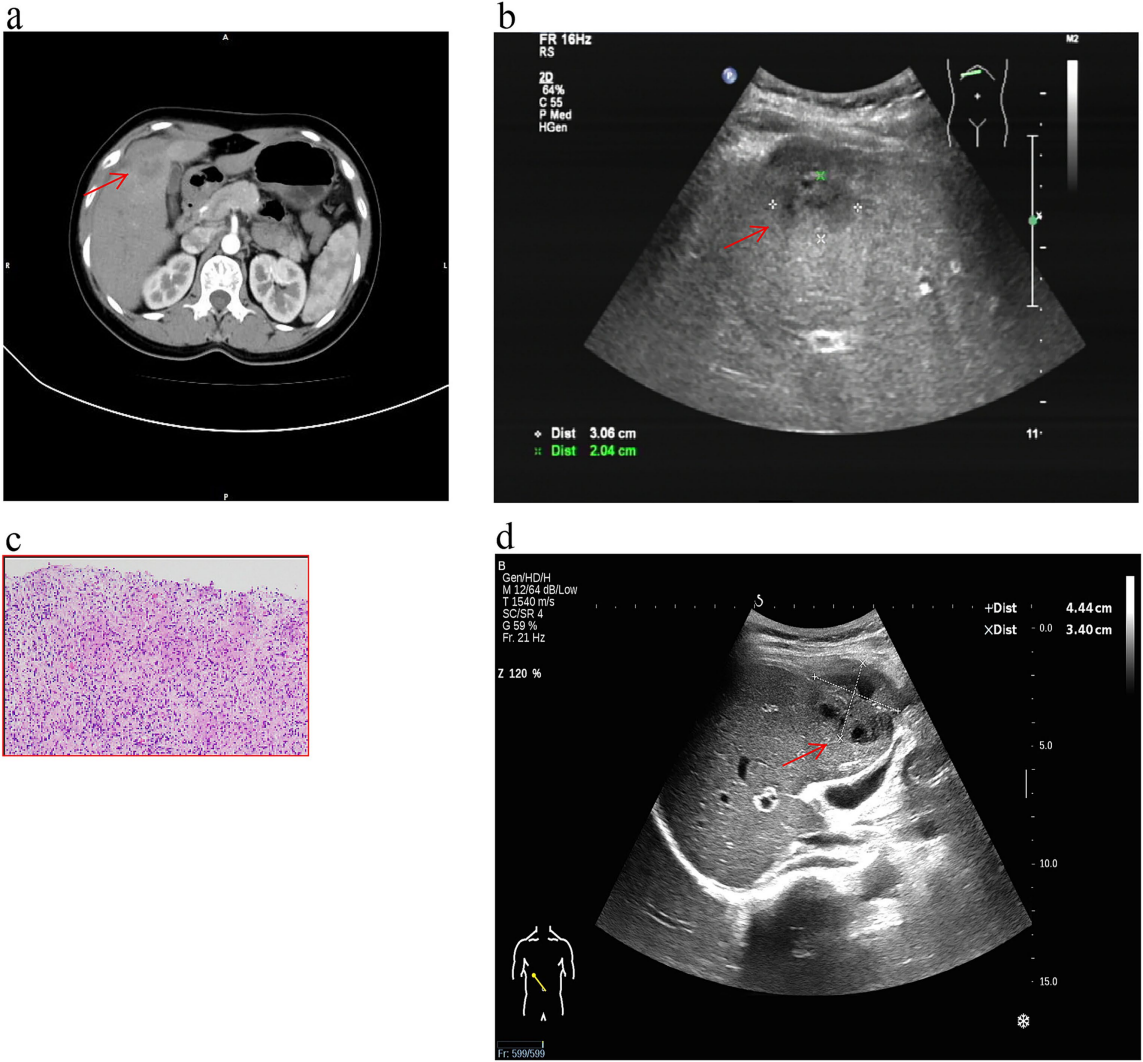
Rare case of tuberculosis liver abscess. **(a)** The abdominal hepatic CT scan revealed a single, well-defined focal lesion measuring 0.8 cm in the low-density area of the right liver lobe and an irregular mass measuring approximately 4.1 cm × 3.1 cm in the right anterior space of the liver (red arrow). **(b)** Ultrasound of the abdomen showed a cystic dark area of 3.7 cm × 2.1 cm in the right posterior lobe of the liver (red arrow). **(c)** Hematoxylin–eosin staining shows chronic granulomatous inflammation suggestive of tuberculosis (HE ×200). **(d)** The abdominal ultrasound reveals a noteworthy enlargement of the abscess, measuring 4.4 cm × 3.4 cm (red arrow).

A 44-year-old woman presented with complaints of abdominal pain for the past 2 months. The patient’s abdominal pain was intermittent, lasting from several minutes to several hours. It was accompanied by a decreased appetite, fever, and night sweats. However, there were no symptoms of nausea, vomiting, changes in bowel movements, chills, or cough with sputum production. The patient was born and currently resides in Luzhou City, Sichuan Province, China. She is a company clerk and denies any history of contact with animals or tuberculosis. She also reports no past medical history of liver disease, tuberculosis, or acquired immune deficiency syndrome (AIDS). In the past year, she had not experienced any weight loss or traveled outside of Luzhou. She was dyspneic (RR = 20 breaths/min), had a temperature of 36.5 °C, a heart rate of 70 beats per minute, blood pressure of 118/78 (normal range, 60–90/90–140) mmHg, and a 33% oxygen saturation on room air at the time of admission. Abdominal examination revealed periumbilical and right lower abdominal wall tenderness and rebound pain. The rest of the system examination was unremarkable. The chest computed tomography (CT) was normal. The contrast-enhanced abdominal CT scan shows a small amount of ascites in the lesser omentum sac and the abdominal and pelvic cavities, with the pelvic cavity being the main site. The greater omentum, mesentery, and peritoneum are extensively thickened, with some areas showing nodular thickening. These are all manifestations of tuberculous peritonitis. The contrast-enhanced CT also reveals a round, low-density, non-enhancing shadow in the right anterior lobe of the liver, with a diameter of approximately 0.8 cm. There is a patchy enhancement in the left medial lobe of the liver during the arterial phase, and the enhancement is consistent with the surrounding liver parenchyma during the venous phase. The hepatic CT showed an irregular mass of approximately 4.1 cm × 3.1 cm in the right anterior space of the liver, part of which invaded the right anterior lobe of the liver (Figure 1a). The findings are consistent with an infection complicated by abscess formation involving the anterior segment of the right hepatic lobe. The right lobe of the liver’s ultrasound revealed a cystic dark region measuring 3.7 cm × 2.1 cm (Figure 1b). To confirm the diagnosis, we performed a liver biopsy (4). Ultrasound-guided biopsy of the lesion was done, which showed chronic granulomatous inflammation suggestive of tuberculosis (Figure 1c). The tuberculin test was positive. TB-DNA real-time PCR performed on the aspirated pus was positive, confirming the presence of *M. tuberculosis* complex DNA. Her total leucocyte count was 7.01 (normal range, 3.5–9.5) × 10^9^/L, neutrophil count was 5.56 (normal range, 1.8–6.3) × 10^9^/L, platelet count was 229 (normal range, 100–350) × 10^9^/L, C-reactive protein (CRP) level was 10.65 mg/L, and erythrocyte sedimentation rate (ESR) was 33 mm^3^. The renal function and electrolyte tests showed urea, 5 (normal range, 3.1–8.0) mmol/L; uric acid, 231 (normal range, 208–428) μmol/L; glomerular filtration rate, 100 (normal range, ≥90) mL/min; creatinine, 72 (normal range, 57–97) μmol/L; potassium, 4.15 (normal range, 3.5–5.3) mmol/L; sodium, 140 (normal range, 137–147) mmol/L; and chloride, 105.2 (normal range, 99–110) mmol/L. The liver function tests showed alanine aminotransferase, 80 (normal range, 9–50) U/L; aspartate aminotransferase, 78 (normal range, 15–40) U/L; total bilirubin, 17 (normal range, 0–26) U/L; direct bilirubin, 8.6 (normal range, 0–8) U/L; indirect bilirubin, 8.4 (normal range, 0–20) U/L; and albumin, 35 (normal range, 40–55) g/L. Blood culture was negative. The carcinoembryonic antigen, CA-19.9, CA-125, and a-fetoprotein serum tumor markers were all within normal limits. The HIV test was negative. She was diagnosed with hepatic tuberculosis and tuberculous peritonitis. Anti-tuberculous medication (isoniazid, rifampin, ethambutol, pyrazinamide) was initiated for the patient. The abscess’s size had increased on the abdominal ultrasonography performed 1 month after the diagnosis (Figure 1d). Abdominal ultrasound revealed a 4.4 cm × 3.4 cm area of heterogeneous echogenicity in the anterior segment of the right hepatic lobe, with ill-defined borders, hypoechoic areas, and internal septations. After discharge, the patient continued to be managed and followed up as an outpatient, but she took herself to a specialized infectious-disease hospital immediately after this repeat ultrasound. During the subsequent period, telephone follow-up indicated that the patient continued anti-tuberculosis therapy (the specific regimen was not documented), with symptoms gradually improving and essentially resolving after 6 months.

## 3 Discussion

Worldwide, tuberculosis still exists. Because of the epidemic of acquired immunodeficiency syndrome, intravenous drug usage, and antibiotic resistance, the incidence of hepatic tuberculosis has grown (5, 6). Of all extra-pulmonary locales, hepatic TB accounts for 3%, and intra-abdominal regions for 9%. Levine (7) divided TB of the liver into five categories: (1) pulmonary TB involving the liver, (2) miliary TB, (3) primary liver TB, (4) focal abscess or tuberculoma, and (5) tuberculous cholangitis. Herein, we describe a case of a 44-year-old woman who presented with abdominal pain, with no prior history of immunodeficiency. A CT scan revealed an abscess in the right lobe of the liver and tuberculous peritonitis. An ultrasound-guided biopsy of the lesion showed chronic granulomatous inflammation, suggesting tuberculosis. The tuberculosis DNA test was positive. Our diagnosis was primarily based on imaging and biopsy results.

Hepatic TB refers to the infection of the liver with *Mycobacterium tuberculosis*, which is clinically rare. Depending on whether there is extrahepatic tuberculosis accompanying the infection of the liver by *Mycobacterium tuberculosis*, it can be divided into primary and secondary (8). Secondary hepatic tuberculosis is usually combined with pulmonary tuberculosis or tuberculous enterocolitis (9). In this case, when hepatic tuberculosis was found, no other definite tuberculosis lesions were found, and it is considered that this case is primary hepatic tuberculosis.

*Mycobacterium tuberculosis* spreads to the liver through hematogenous dissemination. Miliary hepatic tuberculosis is *Mycobacterium tuberculosis* entering the liver through the hepatic artery, and focal hepatic tuberculosis enters the liver through the portal vein; a few can directly invade the liver through the lymphatic system or adjacent lesions (10). Solitary tuberculous liver abscesses are extremely rare and have rarely been described in the literature. *Mycobacterium tuberculosis* does not always form hepatic tuberculosis after invading the liver. Because the liver has a rich blood supply, contains a large number of mononuclear phagocytic cells, and has a strong ability to regenerate and repair, and because bile and the low-oxygen blood of the liver are not conducive to the growth of *Mycobacterium tuberculosis*, it is not easy to form lesions. In this case, the patient did not have an endoscopy scheduled because no symptoms or indicators of enterocolitis were noted during the illness.

Due to the non-specific clinical symptoms and imaging manifestations, hepatic TB is easy to be misdiagnosed (11). Most patients may be asymptomatic, but Esguerra-Paculan and Soldera (12) found that patients may present with symptoms such as hepatomegaly, fever, weakness, night sweats, or abdominal pain (13). Radiological findings can mimic common liver disorders, including tumors, abscesses, or hemangiomata (14). Due to the non-specific clinical features and laboratory test results of hepatic tuberculosis and malignant tumors, it is challenging to distinguish hepatic tuberculosis from malignant tumors, particularly in differentiating it from intrahepatic cholangiocarcinoma. A review of the literature reveals cases where liver abscesses were misdiagnosed as liver tumors and underwent surgical treatment (Table 1). Kale et al. (15) retrospectively analyzed the clinical data of 43 patients at the cancer center and found that one of the imaging characteristics of hepatic tuberculosis is punctate or mass-like calcification. Tumor marker levels can also serve as one of the bases for differential diagnosis, but a definitive diagnosis must rely on pathological examination. Ultrasound/CT-guided percutaneous biopsy has become an important means of reducing unnecessary surgery. This case report describes a case of a tuberculous liver abscess initially presenting with abdominal pain, which was confirmed by liver biopsy. The patient’s tumor marker levels were normal, and the liver CT did not reveal any punctate or mass-like calcifications.

**Table 1:** A summary of the diagnosis of hepatic tuberculosis as a tumor over the past 20 years.

Author	Published time	Diagnose	Location of liver tuberculosis	Challenge	How diagnosed?	Misdiagnosed	Therapy	Past medical history	Outcome
Parsak et al. (6)	2008	Hepatic tuberculosis	Right lobe	Unspecified	Postoperative pathological examination	Liver abscess or infected hydatid disease	Surgical procedures, along with anti-TB	Unspecified	Improved
Parsak et al. (6)	2008	Hepatic tuberculosis	Right lobe	Unspecified	Postoperative pathological examination	Tumor-like mass lesion	Surgical procedures, along with anti-TB	Unspecified	Improved
Landen et al. (17)	2010	Hepatic tuberculosis	Right lobe	Cancer history	Postoperative pathological examination	Colonic liver metastasis	Surgical excision of the lesion	The patient had undergone surgery and adjuvant chemotherapy 2 years ago for Dukes type C colorectal adenocarcinoma	Improved
Wu et al. (13)	2013	Military tuberculous liver abscess	Left lobe	Unspecified	Postoperative pathological examination	Cholangiocarcinoma with intrahepatic metastasis	Surgical procedures, along with anti-TB	The patient had a history of intestinal tuberculosis. HIV status was negative	Improved
Wu et al. (13)	2013	Tuberculous liver abscesses	Hepatic hilar region	Unspecified	Postoperative pathological examination	Hepatic hilar cholangiocarcinoma	Surgical procedures, along with anti-TB	HIV status was negative	Improved
Sharma et al. (11)	2015	Hepatic tuberculosis	Right lobe	Radiological investigations were in favor of biliary cystadenoma	Postoperative pathological examination	Biliary cystadenoma	Surgical excision of the lesion	Unspecified	Improved
Park (18)	2015	Hepatic tuberculosis	Left lobe	Based on the findings from CT and MRI, intrahepatic cholangiocarcinoma is strongly suspected	Postoperative pathological examination	Intrahepatic cholangiocarcinoma	Surgical procedures, along with anti-TB	The patient had no previous history of TB	Improved
Park (18)	2015	Hepatic tuberculosis	Multilobulated liver mass	Based on the findings from CT, MRI, and F-18 FDG PET/CT, intrahepatic cholangiocarcinoma is strongly suspected	Postoperative pathological examination	Intrahepatic cholangiocarcinoma	Surgical procedures, along with anti-TB	The patient had no previous history of TB	Improved
Liu et al. (19)	2017	Hepatic tuberculosis	Caudate lobe	Based on the findings from CT and MRI, a malignant tumor is strongly suspected	Postoperative pathological examination	Intrahepatic carcinoma	Surgical procedures, along with anti-TB	HIV status was negative	Unspecified
Harbi et al. (20)	2018	Hepatic tuberculosis	Hepatic dome	Cancer history	Postoperative pathological examination	Seminoma liver metastasis	Surgical procedures along with anti-TB	An immunocompetent 48-year-old man with a history of non-metastatic seminoma was treated with right orchidectomy followed by 20 Gy radiotherapy	Improved
Maguire et al. (21)	2020	Hepatic tuberculosis	Left lobe	Unspecified	Postoperative pathological examination	Cholangiocarcinoma	Surgical excision of the lesion	She reported no significant past medical history	Improved
Keri et al. (22)	2020	Hepatosplenic tuberculosis	Both the lobe and the spleen	Based on the findings from contrast-enhanced CT, a malignant tumor is strongly suspected	Ultrasound-guided liver biopsy and GeneXpert examination	Malignancy	Anti-TB	Diabetes mellitus and hypertension	Improved
Yang et al. (23)	2020	Hepatosplenic tuberculosis	Left lobe	Both US and MRI findings supported a hepatocellular carcinoma tumor	Postoperative pathological examination	Small hepatocellular carcinoma	Surgical excision of the lesion	He denied any medical history of hepatitis or tuberculosis	Improved
Bova et al. (24)	2023	Hepatosplenic tuberculosis	Caudal lobe and right lobe	Unspecified	Ultrasound-guided liver biopsy	Cholangiocarcinoma	Anti-TB	Unspecified	Improved

Once diagnosed with hepatic tuberculosis, anti-tuberculosis treatment should be started immediately, and anti-tuberculosis treatment should also be started as early as possible if there is strong suspicion of hepatic tuberculosis (5). Early combination therapy with multiple drugs can bring relatively good results; however, delayed diagnosis may lead to liver failure or even death. Treatment for hepatic tuberculosis can involve drug-based anti-tuberculosis and surgical drainage. Drug therapy often uses a regimen primarily consisting of isoniazid, rifampicin, pyrazinamide, and ethambutol (13, 16). A review of the literature suggests that when the effects of anti-tuberculosis treatment are poor, surgical drainage is recommended (13). In this case, the increase in abscess size could be attributed to either treatment failure or an effective therapeutic response accompanied by liquefaction and fibrous septation within the lesion. At the outpatient follow-up, the patient reported less abdominal pain and liver-function tests remained within normal limits, while ultrasound showed a modest increase in abscess size (from 3.7 cm × 2.1 cm to 4.4 cm × 3.4 cm) with indistinct margins, a few hypoechoic areas and internal septations, findings that—taken together with her improved symptoms and biochemistry—lead us to infer that therapy is effective. Unfortunately, she did not remain under our care or follow-up, precluding verification of this inference and complete data acquisition.

## 4 Strengths and limitations

Isolated hepatic tuberculosis in an immunocompetent patient is extremely rare. We conducted a systematic review of cases misdiagnosed and subjected to surgical treatment over the past two decades, revealing a worldwide pattern of unnecessary surgery resulting from such misdiagnosis and underscoring the clinical importance of early biopsy. Nevertheless, the present case report has limitations: comprehensive long-term follow-up data are lacking, and details of subsequent treatment, imaging follow-up, and outcome were obtained only by telephone interview. Despite these constraints, our report highlights the risk of misdiagnosis—a message that retains significant clinical value.

## 5 Conclusion

Hepatic tuberculosis is relatively rare in clinical practice, and some patients lack or omit a history of tuberculosis during consultation. Coupled with atypical clinical manifestations, relying solely on imaging examination results may lead to misdiagnosis and even unnecessary surgery. Tuberculous liver abscesses pose a challenge to the clinical acuity of treating physicians. When encountering suspicious patients in the clinic, a thorough inquiry about the history of tuberculosis should be conducted, and an ultrasound-guided liver lesion biopsy for pathological examination should be performed as early as possible to achieve an early diagnosis and timely treatment.

## Data Availability

The raw data supporting the conclusions of this article will be made available by the authors, without undue reservation.
